# Generalizability of 3D CNN models for age estimation in diverse youth populations using structural MRI

**DOI:** 10.1038/s41598-023-33920-7

**Published:** 2023-04-27

**Authors:** Sergio Leonardo Mendes, Walter Hugo Lopez Pinaya, Pedro Mario Pan, Andrea Parolin Jackowski, Rodrigo Affonseca Bressan, João Ricardo Sato

**Affiliations:** 1 Center of Mathematics, Computing, and Cognition, Universidade Federal Do ABC, Rua Arcturus N. 03, São Bernardo Do Campo, SP 09606-070 Brazil; 2 Department of Biomedical Engineering, King’s College London, London, SE1 7EH UK; 3 Escola Paulista de Medicina, Universidade Federal de São Paulo, R. Maj. Maragliano (UNIFESP), 241—Vila Mariana, São Paulo, SP 04017-030 Brazil; 4 Department of Education, ICT and Learning, Østfold University College, Halden, Norway

**Keywords:** Computational neuroscience, Computational science, Information technology

## Abstract

Recently, several studies have investigated the neurodevelopment of psychiatric disorders using brain data acquired via structural magnetic resonance imaging (sMRI). These analyses have shown the potential of sMRI data to provide a relatively precise characterization of brain structural biomarkers. Despite these advances, a relatively unexplored question is how reliable and consistent a model is when assessing subjects from other independent datasets. In this study, we investigate the performance and generalizability of the same model architecture trained from distinct datasets comprising youths in diverse stages of neurodevelopment and with different mental health conditions. We employed models with the same 3D convolutional neural network (CNN) architecture to assess autism spectrum disorder (ASD), attention deficit hyperactivity disorder (ADHD), brain age, and a measure of dimensional psychopathology, the Child Behavior Checklist (CBCL) total score. The investigated datasets include the Autism Brain Imaging Data Exchange II (ABIDE-II, *N* = 580), Attention Deficit Hyperactivity Disorder (ADHD-200, *N* = 922), Brazilian High-Risk Cohort Study (BHRCS, *N* = 737), and Adolescent Brain Cognitive Development (ABCD, *N* = 11,031). Models’ performance and interpretability were assessed within each dataset (for diagnosis tasks) and inter-datasets (for age estimation). Despite the demographic and phenotypic differences of the subjects, all models presented significant estimations for age (*p* value < 0.001) within and between datasets. In addition, most models showed a moderate to high correlation in age estimation. The results, including the models' brain regions of interest (ROI), were analyzed and discussed in light of the youth neurodevelopmental structural changes. Among other interesting discoveries, we found that less confounded training datasets produce models with higher generalization capacity.

## Introduction

In the last few decades, several studies have investigated neurodevelopment and psychiatric disorders using brain data acquired via structural magnetic resonance imaging (sMRI)^1–3^. These analyses have shown the great potential of sMRI data as a biomarker^4–6^. One main asset of the current methodology is the ability to perform relatively precise characterization of brain structures, which is essential for using structural neuroimaging data to understand the brain mechanisms of psychiatric disorders^1^. Moreover, sMRI biomarkers are already an important part of clinical assessment for neurodegenerative diseases such as Alzheimer's and other prevalent dementias^7^. Unfortunately, most psychiatric disorders still rely solely on clinical judgment. Therefore, investigations on neuroimaging biomarkers, particularly in youth, may help clinicians in differentiating between typical and atypical developmental trajectories^8^. These quantitative measures could help distinguish typically developing (TD) from children with attention deficit hyperactivity disorder (ADHD)^9^ or autism spectrum disorder (ASD)^10^. Furthermore, these investigations could be useful for understanding the neural basis of dimensional symptoms in psychopathology.

Recent studies have explored typical neurodevelopment based on age estimation from sMRI data and convolutional neural network (CNN) machine-learning models^8,11,12^. Increased brain age estimations have been correlated with an increased risk of schizophrenia, epilepsy, Down’s syndrome, and progression to Alzheimer’s disease in high-risk subjects^13–16^. Notably, decreased predicted age has been correlated with protective influences exerted by meditation, increased education level, and physical exercises^17,18^. In recent years, CNN-based deep learning approaches outperformed previous shallow models (such as Gaussian process regression) in estimating brain age from sMRI^11^, becoming the state of the art for this task^12^. Despite promising results, CNNs (as well as other artificial neural networks) can be difficult to interpret, providing little insight into the nature of the neural mechanisms underlying psychiatric disorders^19,20^. Furthermore, the generalizability and consistency of CNN models across different datasets remain an open question. How a model trained in one dataset performs when evaluating other distinct datasets remains poorly explored, which is critical for the clinical use of any proposed biomarker.

A model trained from a given dataset should perform adequately when estimating new unseen subjects. However, these new subjects do not always meet the same characteristics (i.e., age, sex, ethnicity, or mental health conditions) as those included in the training data. Furthermore, knowing what features a model focuses on when making decisions is essential. That is, which are the most representative ROIs during estimations? Are these ROIs the same, or do they vary when evaluating unseen data? Are the most representative ROIs equal or different for models trained from similar versus distinct populations? The answer to these questions is very relevant when making conclusions for a study. That is, how much the findings can be generalized to similar subjects but with distinct demographic characteristics. To the best of our knowledge, these questions are relatively unexplored in neuroimaging data analyzed via CNNs, especially for the neurodevelopment stage between childhood and adulthood.

The current study investigates the performance and generalizability of models trained from distinct datasets comprising youths in diverse stages of neurodevelopment and with different mental health conditions. We trained 3D CNN models of the same architecture to assess ASD, ADHD, brain age, and Child Behavior Checklist (CBCL) total score, with no previous hypothesis. Then, we evaluated the performance and interpretability of these models within each dataset (for diagnosis tasks) and inter-datasets (for age estimation). Finally, the performance and relevant brain regions of interest were analyzed and discussed in light of neuroscience.

## Materials and methods

### Data description

The studied data were retrieved from two public sets: Autism Brain Imaging Data Exchange II (ABIDE-II) and ADHD-200, and from two large neurodevelopmental studies: the Adolescent Brain Cognitive Development (ABCD) and Brazilian High-Risk Cohort Study (BHRCS)^21–24^. We used only T1-weighted sMRI data from all investigated datasets. For the ABCD and BHRCS datasets, only data from the first collection (i.e., baseline—wave zero) were considered. ABIDE-II and ADHD-200 images were collected from several locations in different countries, including 19 location sites for ABIDE-II, and 8 sites for ADHD-200. Thus, the acquisition parameters of ABIDE-II and ADHD-200 varied, comprising 1.5 T and 3 T scanners, each hosting a head coil from 8 to 32 channels. These public datasets can be found on the ADHD-200 (http://fcon_1000.projects.nitrc.org/indi/adhd200/) and ABIDE-II (http://fcon_1000.projects.nitrc.org/indi/abide/abide_II.html) websites. ABCD data were collected from multi-brand 3 T scanners, from 21 sites in the USA. Additional detailed acquisition parameters can be retrieved from ABCD (https://abcdstudy.org/images/Protocol_Imaging_Sequences.pdf). The BHRCS data were collected in two Brazilian cities using GE Signa HDX 1.5 T and GE Signa HD 1.5 T scanners. Detailed acquisition parameters for the BHRCS can be found in a study by Sato et al.^25^. Data were collected and made available according to guidelines and approval from the local ethics committee of each project.

### Subjects

**Figure 1:**
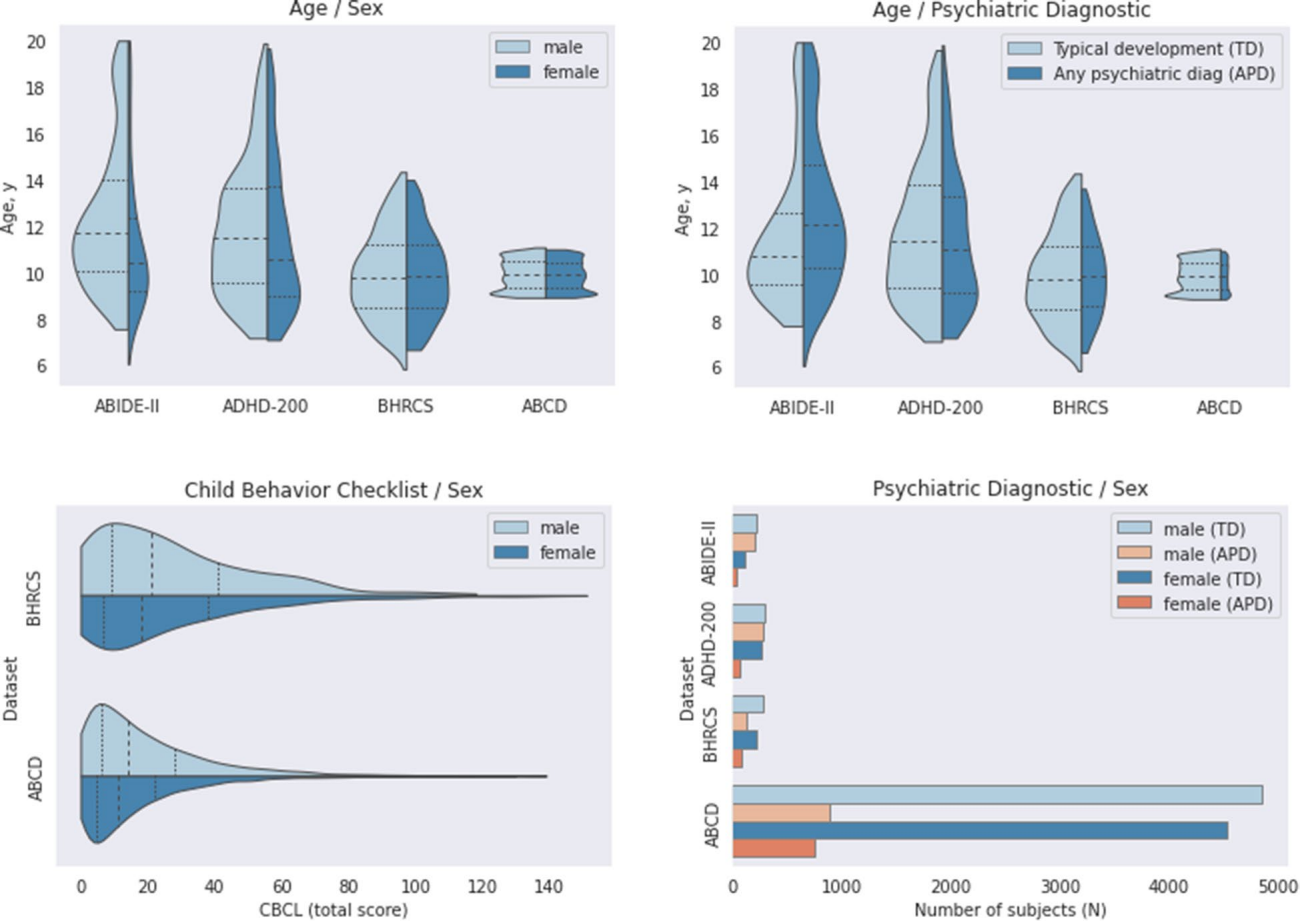
Demographic and phenotypic distribution of subjects. In violin plots, the dotted lines show the quartiles. Ages are presented in years, and CBCL in raw values. Acronyms: TD = typical development, APD = any psychiatric diagnostic, and CBCL = child behavior checklist. APD indicates autism spectrum disorder for ABIDE-II, attention deficit hyperactivity disorder for ADHD-200, and any psychiatric diagnostic (from DSM-IV or DSM-V) for BHRCS and ABCD, respectively.

As we focused on the study of neurodevelopmental processes in youth, we selected only subjects younger than 20 years of age from all included datasets. Some participants had more than one sMRI scan within the dataset (from different scanning sessions). We only used the earliest sMRI from each subject in these cases. Data without information on sex, age, or psychiatric evaluation (i.e., TD, ASD, ADHD, or CBCL) were discarded. Furthermore, each subject belonged exclusively to a single dataset. That is, there is no overlapping diagnosis of subjects included in different models due to having multiple disorders (i.e., ASD and ADHD). After this filtering, we arrived at the following sample sizes: ABIDE-II (*N* = 580), ADHD-200 (*N* = 922), BHRCS (*N* = 737), and ABCD (*N* = 11,031). Figure 1 shows the demographic and phenotypic overview of the study data.

### MRI processing

The sMRI images were processed using VBM^26^ via the Statistical Parametric Mapping software^27^ (SPM12 v7771, from https://www.fil.ion.ucl.ac.uk/spm/software/spm12/). VBM spatially normalizes MRI images to the same stereotactic space, allowing the extraction of different brain tissues from images partitioned with correction for nonuniform intensity variations^26^. The processing steps followed the recommended script for VBM, as follows:

First, sMRI data were spatially segmented to segregate grey matter (GM), white matter (WM), and cerebrospinal fluid (CSF)^28^. In this step, the skull, tissues, and artifacts outside the brain were removed from the original image. Second, the DARTEL algorithm^29^ was applied to increase the accuracy of inter-subject alignment. This transformation works by aligning GM among the images, while simultaneously aligning WM during the generation of a template to which the data are iteratively aligned^30^. Third, the resulting files from the previous step were spatially normalized, Jacobian scaled, and smoothed with the Gaussian full width at half maximum (FWHM) set to 8 mm to generate images in the Montreal Neurological Institute (MNI) coordinate system^31,32^.

After the transformations, each sMRI produced three 3D matrices (i.e., GM, WM, and CSF), with each voxel carrying the probable density of brain tissue at that location. The produced matrixes with a dimension of 121 × 145 × 121 (voxel size = 1.5 mm) were padded and trimmed resulting in 128 × 128 × 128 volumes. This transformation affected only background voxels (outside the brain) and was applied for best GPU usage (https://www.tensorflow.org/guide/gpu_performance_analysis). The processing was done in separate batches of tasks (i.e., one batch per dataset) to ensure that there would be no bias due to the interaction of examples in different datasets.

We used only the GM and WM resulting data, and all voxels outside the brain were set to zero. This step was conducted to ensure that only data related to brain tissues (i.e., neurodevelopment data) would be available to the models. Despite the potential that out-of-brain data has to improve the accuracy of models, this information could add confounding variables to the analyses. Therefore, we opted for potentially worse performance in favor of more interpretable and reliable results.

### Evaluation procedure

We trained CNN models from different datasets to perform several tasks: to classify the mental health status (i.e., TD, ASD, and ADHD), estimate the CBCL total score via regression, and estimate brain age via regression. For each dataset, the partitions for training, validation, and testing were created from a nested cross-validation scheme, where the outer cross-validation was a k-fold, and the inner cross-validation was a random split of 90% for training and 10% for validation. Therefore, we had the advantage of robust nested cross-validation while preserving the lower processing time of a non-nested schema^8^. The ABIDE-II, ADHD-200, and BHRCS datasets were assessed in K = 5 folds, while the ABCD was evaluated in K = 10 folds. Unlike other datasets, the huge sample size of ABCD (*N* = 11,031) allowed the use of 10 folds, maximizing the sample size of the training sets and still guaranteeing large test samples. All the partitions’ splits were stratified by sex and age. As age is a continuum variable, before stratification, we discretized the distribution in 15 categorical quantiles. As there were few subjects with higher CBCL scores or positive diagnoses for ASD or ADHD (see Fig. 1), additional stratifications by CBCL, ASD, or ADHD were not feasible and therefore were not performed.

The validation set allowed the extraction of metrics for model selection, and the test set remained unseen until the models were fully trained. Therefore, the performance metrics were assessed from unbiased and unexplored data according to the following schema:

1. AGE regression models (ABIDE-II, ADHD-200, BHRCS, and ABCD) were evaluated on their respective test sets.
2. The best-performing AGE model from one dataset was evaluated on the full independent (out-of-sample) datasets.
3. CBCL regression models (BHRCS and ABCD) were evaluated on their respective test sets.
4. ASD classification models (ABIDE-II) were evaluated on their respective test sets.
5. ADHD classification models (ADHD-200) were evaluated on their respective test sets.

The fitting of additional age models from full training datasets to evaluate the out-of-sample data could improve performance. However, we intended to compare the results of the same trained model within and between datasets. Accordingly, we chose the best-performing age model from cross-validation to evaluate the external datasets, potentially losing performance in favor of comparability. In addition, using a k-fold split in the inner loop of the cross-validation (instead of a training/validation split) followed by creating an ensemble of the inner models (to evaluate the test set) could increase robustness and generalizability. However, this approach would increase the training times by 25 to 50 times, so we opted not to implement this strategy.

To evaluate the models’ performance for the regression tasks, we assessed MAE (mean absolute error), Pearson’s correlation, P-value of the Pearson’s correlation, and the prediction R^2^ (also known as cross-validation R^2^ or q^2^, which best assesses numerical accuracy for regression tasks^33^). For the classification tasks, we assessed sensitivity, specificity, balanced accuracy (mean between sensitivity and specificity), and area under the receiver operating characteristic curve (AUC). We chose balanced accuracy (instead of simple accuracy) because it can better evaluate unbalanced data, which can bias the models toward classifying minority cases into majorities^34^. To find the best cutoff values, we used a ROC operating point selection that maximizes the harmonic mean between the sensitivity and specificity^34^. Thus, for each trained model, validation data was used to find an optimal cutoff, and then, this value was used to classify the new test data. We ran permutation tests (with 1,000 permutations) to determine the *p* values for the classification predictions. The accepted statistical significance level (alpha) was 5%.

We adopted the approach proposed by Dinga et al.^35^ to assess the effects of confounding variables, which uses trained model predictions to estimate confounding effects. For that, three different models are fitted to the target: (1) using only confounders as predictors, (2) using only predictions as predictors, and (3) using confounders and predictions as predictors. Next, the coefficient of determination (R^2^ for regression and D^2^ for classification) is calculated for each model. Then, the results are separated into the contributions from predictions only, confounders only, and shared (i.e., predictions + confounders). This method is reliable even when other methods (e.g., methods based on input variable adjustment) fail^35^. The confounders selected for age predictions were: sex, acquisition site, and total brain volume. For ADHD, ASD, and CBCL estimations, the chosen confounders were: age, sex, acquisition site, and total brain volume.

### Model architecture and training

The model architecture used in this study was projected by Cole et al.^11^. This architecture was chosen because: (1) it was designed to predict brain age with satisfactory performance, and (2) it was not created or optimized to any of the studied datasets (i.e., ABIDE-II, ADHD-200, BHRCS, or ABCD). Therefore, the model architecture had no performance bias toward any of the evaluated datasets.

In summary, the model architecture contains five blocks. Each block is composed of: a (3 × 3 × 3) convolutional layer (stride = 1), rectified linear unit (ReLU), (3 × 3 × 3) convolutional layer (stride = 1), 3d batch normalization layer^36^, ReLU and finally a (2 × 2 × 2) max-pooling layer (stride = 2)^11^. The number of channels was set to eight in the first block and doubled after each max-pooling layer to obtain a sufficiently rich brain representation^11^. The final prediction is obtained after applying a fully connected layer, which maps the output of the last block to a single output value^11^. The original study does not state what value was set for L2 regularization. Therefore, to prevent overfitting, we adopted L2 kernel regularizers (equal to 0.001) in every convolutional and fully connected layer, as done in a related study^8^. We also padded and trimmed the brain input matrix, which originally had a size of 121 × 145 × 121 × 2, to a size of 128 × 128 × 128 × 2, aiming for memory optimization and training performance^8^. The final output layer was chosen according to the model task. For the regressions (*i.e*., AGE or CBCL), we used the ReLU activation with mean squared error (MSE) loss function. For the classifications (i.e., TD, ASD, or ADHD), we used sigmoid activation with binary cross-entropy as the loss function.

Adam algorithm was chosen to optimize the objective loss^37^. Briefly, this is a gradient-based method that employs adaptive learning rates. Adam’s initial learning rate was set to 0.001, and the exponential decay rates for the first and second estimate moments were maintained at their default values (i.e., 0.9 and 0.999, respectively). The batch size was set to 48 examples. The examples were not stratified at the batch level, and they were randomly shuffled before batch splits. The number of epochs was set to 1000, and an early stopping technique was adopted to stop the training process when there was no improvement in the validation output loss for 75 consecutive epochs^8^. In addition, we used a technique named model checkpoint, where the model is evaluated against its validation set after every epoch, and the best-performing model weights are saved. This strategy can prevent overfitting by storing the weights at an optimal moment during the training.

### Models’ interpretability

To address the low interpretability level of neural networks that provide little or no insight into the nature of data^19,20^, we used SmoothGrad^8,38^. This algorithm generates a sensitivity map of voxels that contributes the most to the neural network decisions. It measures the impact that small perturbations in the input images produce in the output gradients. Although it is similar to other algorithms (e.g., Vanilla Saliency^39^), SmoothGrad produces sharper pictures due to its strategy of averaging results from different noise patterns applied to every input picture^38^.

Sensitivity map algorithms often produce gradients with signed values^38^. However, there is ambiguity in converting these signed values to visualization colors, as the gradient direction is context-dependent^8^. To resolve this issue, we adopted the absolute values of the gradients, which can produce clearer pictures^8,38,40^. Following the SmoothGrad authors^38^, we set the noise level to 20% and the number of noisy samples to 50. The implementation used by this study is available in an open-source library named tf-keras-vis (available at https://pypi.org/project/tf-keras-vis).

The attention maps were generated from (1) the test sets corresponding to each of the k-fold cross-validations and (2) the full out-of-sample (independent) tested datasets. For the cross-validation test sets, the results were first averaged within each fold, and then normalized and averaged across all folds. This resulted in (1a) one attention map for each dataset and model task (i.e., predicting age or mental health status), and (2a) one attention map for each of the full out-of-sample tested datasets. This strategy captures common brain structures that are most descriptive for the models’ decision-making^8^. Finally, the resultant attention maps were intersected with the AAL3 3D brain atlas^41^ to provide ROI identifications and then rendered in the MRICron software (https://www.nitrc.org/projects/mricron) to provide 3d visualization of brain locations^8^.

### Software and hardware specification

The sMRI preprocessing was done through the SPM12 v7771 software (https://www.fil.ion.ucl.ac.uk/spm/software/spm12/). All further steps used Python 3.8.5 and Tensorflow 2.4.0 (https://docs.nvidia.com/deeplearning/frameworks/tensorflow-release-notes/rel_21-03.html). The machine learning experiments were performed on an NVIDIA DGX-2 server, within a Docker virtual machine containing 4 CPUs @2.7Ghz and 1 GPU TESLA V100-SXM3-32 GB. All source codes are available at Github (https://github.com/SergioLeonardoMendes/3dcnn_smri_generalization).

## Results

Demographic and phenotypic analyses showed distinct distributions of sex, age, and/or mental health conditions for each dataset (see Table 1). For all datasets, the models trained to predict age were able to learn, showing statistically significant correlations between the predicted and target ages (i.e., *r* >  = 0.45 and *p* values < 0.001). Moreover, the best-performing age model from each dataset cross-validation was able to generalize well to other (independent) datasets (correlation *p* values < 0.001). However, the models trained to predict CBCL were not able to adequately estimate dimensional psychopathology using CBCL’s total score. That is, the estimations of CBCL in BHRCS and ABCD datasets were not statistically significant (*p* values: 0.20 and 0.07). Regarding discrete psychiatric diagnoses, models trained from ABIDE-II to classify ASD presented poor performance (*p* value = 0.53, AUC = 0.48 ± 0.09), but models trained from ADHD-200 to classify ADHD were able to learn, achieving above chance metrics (*p* value = 0.02, AUC = 0.64 ± 0.04, specificity = 0.62 ± 0.04, sensitivity = 0.59 ± 0.12, balanced accuracy = 0.60 ± 0.04).

**Table 1:** Subjects’ demographic and phenotypic information.

Dataset	N	Female, %	Age, y ± SD	Age range, y	AMD, %	CBCL ± SD
ABIDE-II	580	26.2%	12.1 ± 3.2	6.1–20.0	43.3%	–
ADHD-200	922	36.9%	11.7 ± 3.0	7.1–19.9	38.7%	–
BHRCS	737	42.9%	9.9 ± 1.9	5.8–14.3	30.5%	27.1 ± 25.2
ABCD	11,031	48.0%	9.9 ± 0.6	8.9–11.1	15.0%	18.1 ± 17.9

The sample size (N) is shown in numbers. Age is in years ± standard deviation and range of minimum–maximum years of age. The CBCL total score is a raw scale. Subjects with Any Mental Disorder are grouped in AMD. For ABIDE-II, AMD contains subjects with different levels of the autism spectrum, while ADHD-200 AMD includes different subtypes of ADHD. For both BHRCS and ABCD datasets, AMD comprises subjects with at least one diagnostic of mental disorders according to DSM-IV (for BHRCS) or DSM-V (for ABCD).

Out of the age models, the ones trained from ADHD-200 achieved the best correlation and coefficient of determination in cross-validation (*r* = 0.84 ± 0.02 and prediction *R*^*2*^_*cv*_ = 0.62 ± 0.14). When considering the metric MAE, the ABCD models performed best in age cross-validation (MAE = 0.47 ± 0.01 years). Using correlation as a metric to assess generalization capacity, the ABCD model evaluated on ADHD-200 presented the best result (*r* = 0.80). All assessed metrics are presented in Tables 2 and 3.

**Table 2:** Regression performance metrics.

AGE regression cross-validation	n	MAE, y	r	*r p* value	R^2^_cv_
ABIDE-II model, fivefold CV on test set	116	1.51 ± 0.18	0.81 ± 0.02	< 0.001	0.62 ± 0.09
ADHD-200 model, fivefold CV on test set	184	1.41 ± 0.25	0.84 ± 0.02	< 0.001	0.62 ± 0.14
BHRCS model, fivefold CV on test set	147	1.22 ± 0.15	0.62 ± 0.11	< 0.001	0.35 ± 0.13
ABCD model, tenfold CV on test set	1103	0.47 ± 0.01	0.45 ± 0.02	< 0.001	0.18 ± 0.04

AGE regression cross-data set evaluation	n	MAE, y	r	*r p* value	R^2^
ABIDE-II model on ADHD-200 full data	922	1.88	0.71	< 0.001	0.32
ABIDE-II model on BHRCS full data	737	2.57	0.50	< 0.001	− 1.7
ABIDE-II model on ABCD full data	11,031	1.98	0.27	< 0.001	− 13.5
ADHD-200 model on ABIDE-II full data	580	1.56	0.76	< 0.001	0.56
ADHD-200 model on BHRCS full data	737	1.44	0.53	< 0.001	0.0
ADHD-200 model on ABCD full data	11,031	1.29	0.31	< 0.001	− 5.79
BHRCS model on ABIDE-II full data	580	1.74	0.72	< 0.001	0.43
BHRCS model on ADHD-200 full data	922	1.59	0.75	< 0.001	0.49
BHRCS model on ABCD full data	11,031	0.92	0.30	< 0.001	− 2.43
ABCD model on ABIDE-II full data	580	2.26	0.65	< 0.001	− 0.07
ABCD model on ADHD-200 full data	922	2.17	0.80	< 0.001	0.07
ABCD model on BHRCS full data	737	1.47	0.56	< 0.001	0.12

CBCL Regression cross-validation	n	MAE, y	r	*p* value	R^2^
BHRCS model, fivefold CV on test set	147	19.3	0.09	0.20	− 0.01
ABCD model, tenfold CV on test set	1103	13.2	0.08	0.07	− 0.01

The performance indicators are presented in mean values ± standard deviation. The chosen model for between dataset evaluation is the best-performing from the cross-validation. In column titles, **n** is the sample size, **MAE** is the mean absolute error, **r** is the Pearson’s correlation between prediction and target, and **R**^**2**^ is the prediction R^2^ (also known as cross-validation R^2^ or q^2^). While **r** shows how correlated predictions and targets are, **R**^**2**^ expresses how much of the target’s variability can be explained by predictions. Note that good MAEs (low values) may present poor correlations due to a narrow age range in the evaluated dataset. *p* values lower than 0.001 were omitted for clarity. *CBCL* Child behavior checklist total score.

**Table 3:** Classification performance metrics.

ASD/ADHD classification cross-validation	n	Specificity	Sensitivity	Bal. acc	Auc	*p* value
ASD: ABIDE-II model, fivefold CV on test set	116	0.50 ± 0.34	0.46 ± 0.41	0.48 ± 0.07	0.48 ± 0.09	0.53
ADHD: ADHD-200 model, fivefold CV on test set	184	0.62 ± 0.04	0.59 ± 0.12	0.60 ± 0.04	0.64 ± 0.04	0.02

The performance metrics are in mean values ± standard deviation. *ASD* Autism spectrum disorder, *ADHD* Attention deficit hyperactivity disorder.

The confounding analysis was conducted as planned, generating the metrics in Table 4. For age predictions, low confounding effects were observed in the BHRCS (∆confounds = 0.07, shared < 0.01) and ABCD (∆confounds = 0.04, shared = 0.02). However, moderate to high confounding effects were observed in ADHD-200 (∆confounds = 0.05, shared = 0.40) and ABIDE-II (∆confounds = 0.13, shared = 0.55). For ADHD classification, almost all the performance can be explained by confounders (∆predictions = 0.01, ∆confounds = 0.22, shared = 0.03). Only models performing above-chance predictions had their confounders evaluated (i.e., the ASD and CBCL models were ignored).

**Table 4:** Confounding effects for models’ predictions.

Dataset, task	∆Confounds	∆Predictions	Shared
ABIDE-II, AGE predictions (fivefold test sets)	0.13 ± 0.04	0.11 ± 0.02	0.55 ± 0.01
ADHD-200, AGE predictions (fivefold test sets)	0.05 ± 0.01	0.31 ± 0.03	0.40 ± 0.05
BHRCS, AGE predictions (fivefold test sets)	0.07 ± 0.04	0.39 ± 0.11	0.00 ± 0.02
ABCD, AGE predictions (tenfold test sets)	0.04 ± 0.01	0.19 ± 0.02	0.02 ± 0.01
ADHD-200, ADHD predictions (fivefold test sets)	0.22 ± 0.04	0.01 ± 0.02	0.03 ± 0.03

Metrics are presented in mean values ± standard deviation. The numbers reflect the coefficient of determination of the target (R^2^_cv_ for AGE and D^2^_cv_ for ADHD). In column titles, ∆Confounds = confounds only, ∆Predictions = predictions only, and Shared = both confounds and predictions.

**Figure 2:**
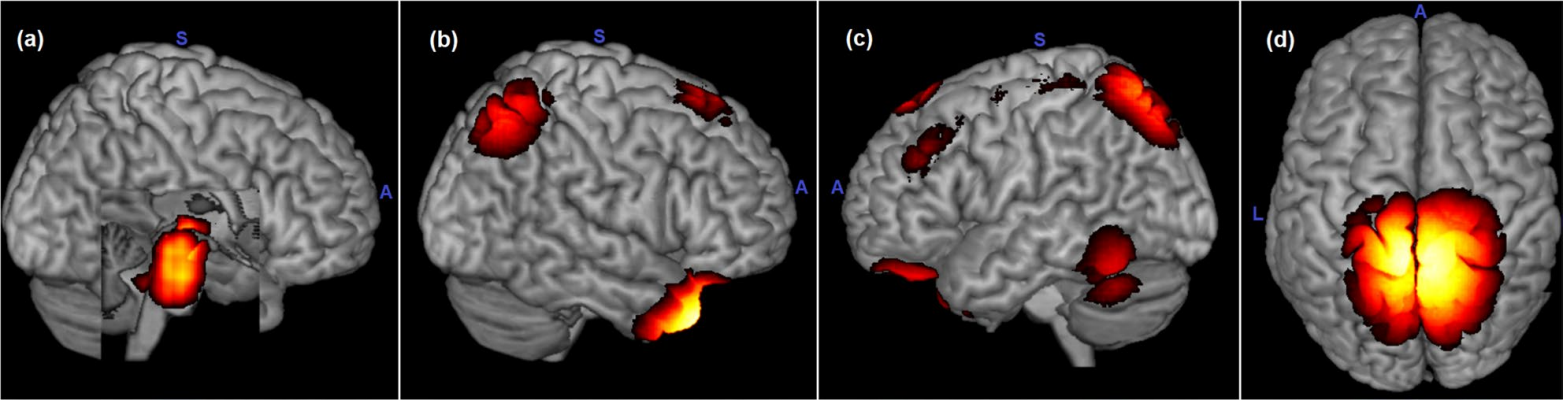
Top representative regions of age models. The images reflect models trained from (**a**) ABCD, (**b**) BHRCS, (**c**) ADHD-200, and (**d**) ABIDE-II. The attention maps were averaged from all cross-validation models. Note that ABCD attentions are subcortical regions. Acronyms: L = left, A = anterior, and S = superior.

By analyzing the top 10 most representative ROIs for age estimation, we found that the ABCD included the substantia nigra pars compacta and pars reticulata (left and right), red nucleus (left and right), ventral tegmental area (left and right), and raphe nucleus (dorsal and median). For ABIDE-II, the ROIs that arose were the paracentral lobules (left and right), superior parietal gyrus (left and right), inferior parietal gyrus (right), precuneus (left and right), postcentral gyrus (right), superior occipital gyrus (gyrus), and motor supplementary cortex (right). In the ADHD-200, the ROIs that emerged were the medial orbital gyrus (left and right), anterior orbital gyrus (left), gyrus rectus (left and right), middle temporal gyrus (left and right), inferior temporal gyrus (left), superior parietal gyrus (left), and angular gyrus (left). Interestingly, all the top ROIs of BHRCS were in the right side of the brain, and these regions included the temporal gyrus (superior and middle), orbital gyrus (anterior, posterior, medial, and lateral), parietal gyrus (superior and inferior), angular gyrus, and inferior frontal gyrus (opercular part) (Fig. 2).

**Figure 3:**
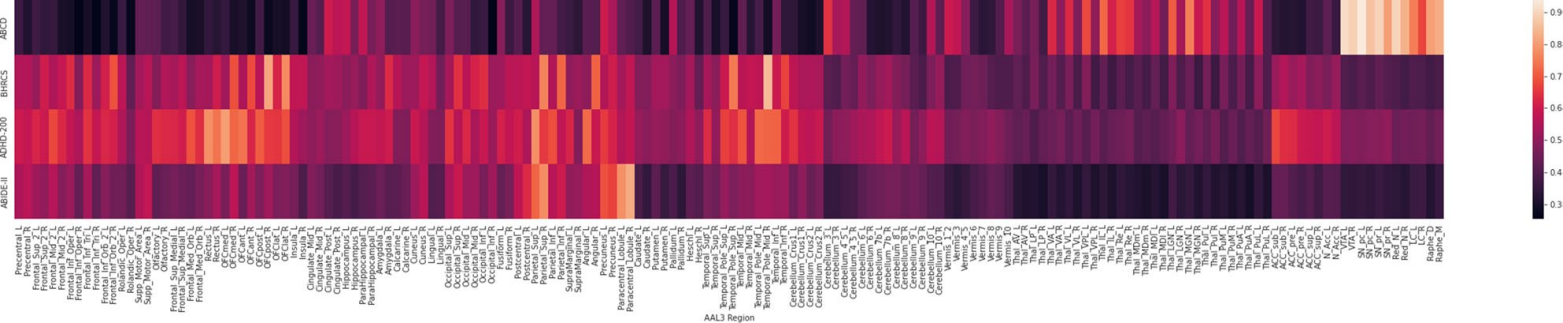
Heat map of importance for age models within each dataset. Lighter areas indicate more representative brain regions. The datasets and ROIs are in the y- and x-axis, respectively. The ROIs follow the AAL3 atlas acronyms^41^.

To better illustrate the distribution of ROIs’ representativeness for age models, Fig. 3 depicts the un-thresholded attention maps for all brain regions within each dataset.

For ADHD classification in ADHD-200 data, the top ROIs were the superior parietal gyrus (left), middle frontal gyrus (left), superior occipital gyrus (right), parahippocampal gyrus (right), angular gyrus (right), amygdala (right), ventral tegmental area (right), median raphe nucleus, locus coeruleus (right), and substantia nigra pars compacta (right). However, the confounding effects (see Table 4) lead us to believe that these ROIs are mostly related to confounders (i.e., age, sex, acquisition site, and total brain volume) rather than ADHD.

**Figure 4:**
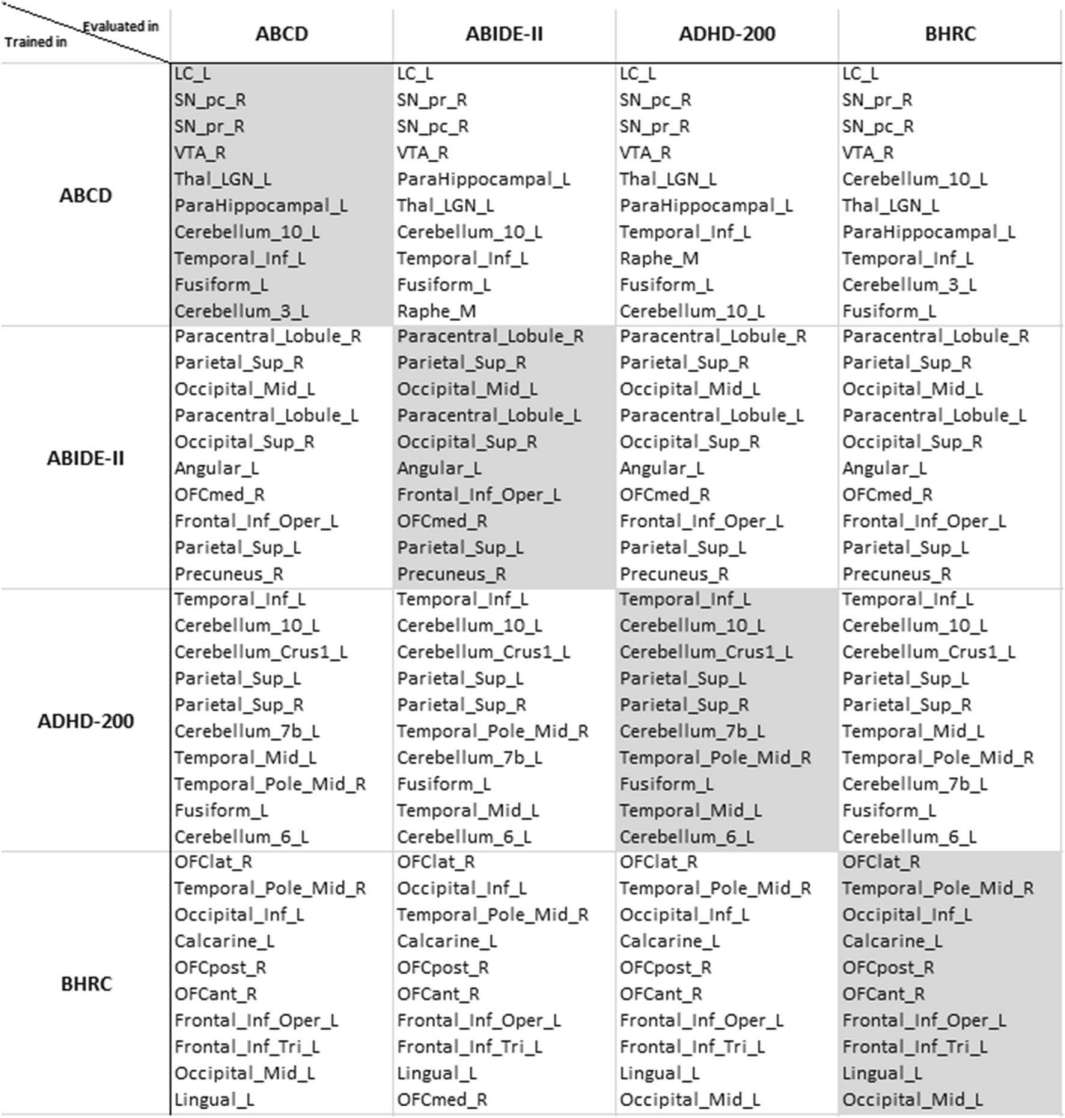
Top 10 most representative ROIs for different datasets in age prediction. The lists are ordered by the most to the less representative ROI. Notice that the set of ROIs from a given trained model is invariant to different datasets, and there is little or no difference in the ROIs’ importance for the evaluated datasets. The chosen trained models are the ones with the best performance in cross-validation.

To investigate the generalization process, we used the best-performing age model from each dataset to extract the top representative ROIs for the out-of-sample datasets. For ABIDE-II and ADHD-200 models, the most representative ROIs were the same for all evaluated datasets. For ABCD and BHRCS models, almost all ROIs (9 out of 10) were identical in all datasets. In other words, the set of most representative ROIs of each model was invariant to different evaluated datasets. The list of ROIs is presented in Fig. 4, following the AAL3 acronyms^41^.

## Discussion

Each studied dataset presents specific characteristics, making it unique in terms of demographic and phenotypic distribution. Each set has a unique distribution of sex, age, ethnicity, and mental health conditions (i.e., levels of total CBCL, ASD, ADHD, and TD). Moreover, the datasets are composed of images collected from different sites, from multiple scanner brands and models, presenting distinct parameter settings. Despite these differences, the models trained to estimate age were able to (1) show good performance in their test sets, (2) generalize reasonably well to out-of-sample datasets and (3) present almost identical brain ROIs for the out-of-sample dataset evaluations. However, the models trained to predict CBCL total scores were not able to learn from BHRCS (*p* value = 0.20) and ABCD (*p* value = 0.07). Models trained to detect ASD also showed below-chance prediction (*p* value = 0.53), while models trained to detect ADHD had above-chance performance (*p* value = 0.02). However, posterior statistical analyses revealed that both ADHD and ASD predictions were almost exclusively influenced by confounders (see Table 4). Therefore, the brain structural differences produced by ADHD, ASD, and dimensional psychopathologies assessed by CBCL total score were not captured by the sMRI in association with the CNN methods employed in this study.

Comparing performance among different studies is not a trivial task, as different studies commonly use distinct methods, preprocessing steps, and criteria for including participants. However, the performance of our study seems to be in line with the modern related literature. A recent study used a normative boosting model trained from data combining six datasets (including ABIDE-II) to predict the age of adolescents, resulting in MAE = 1.53 years for typical development, and MAE = 1.49 for at-risk individuals^42^. This aligns with the results we obtained for the ABIDE-II cross-validation (MAE = 1.51). Another study used multitask learning CNN models^8^ to predict age, obtaining correlations very similar to those achieved here for the cross-validations of ABIDE-II (r = 0.76 vs r = 0.81), and for ADHD-200 (r = 0.84 vs r = 0.84). Furthermore, the multitask learning study^8^ achieved similar correlations for the ABIDE-II model predicting ADHD-200 (r = 0.72 vs r = 0.71), and for the ADHD-200 model predicting ABIDE-II (r = 0.75 vs r = 0.76). Aside from these interesting findings, to the best of our knowledge, no studies in the current literature evaluate the between-dataset performance of ABCD and BHRCS.

Interestingly, our models’ capacity to estimate age presented statistically significant performances for distinct out-of-sample datasets, even considering the narrow age ranges of ABCD and BHRCS (see Fig. 1). This is evidenced by analyzing the mean of the correlations obtained by each model on the out-of-sample datasets (see cross-dataset evaluation in Table 2). The means of correlations presented by the models on cross-dataset evaluations were: ABCD (r_mean_ = 0.67), BHRCS (r_mean_ = 0.59), ADHD-200 (r_mean_ = 0.53), and ABIDE-II (r_mean_ = 0.49). Interestingly, the less confounded models ABCD (∆confounds = 0.04, shared = 0.02) and BHRCS (∆confounds = 0.07, shared < 0.01) presented better generalization capacity than that of the more confounded ones, ADHD-200 (∆confounds = 0.05, shared = 0.40) and ABIDE-II (∆confounds = 0.13, shared = 0.55). These indicate that less confounded datasets may push the models to learn more robust features (i.e., not related to confounders), which results in better generalization capacity for out-of-sample datasets.

Other unexpected findings come from the observation that the model trained from ABCD (with the narrowest age range) presented the best cross-dataset correlation (r = 0.8) on ADHD 200 (with a wider age range). This may have occurred due to some characteristics of the studied datasets. The ABCD has a large sample size, being more than 10 times bigger than the other studied datasets (see section "Subjects"). Whereas small sample sizes tend to deliver better accuracies (within the dataset), large sample sizes present better generalization power^43^. Moreover, the ABCD is the least confounded of the studied datasets, which we postulate results in better generalization capacity. The observation of the distributions for age (see Fig. 1), lead us to suppose that ABCD models should perform best on the BHRCS dataset. However, the ABCD and BHRCS datasets were exclusively collected by 3 T scanners (ABCD) and 1.5 T scanners (BHRCS). We guess the differences in scanners’ acquisition parameters may have contributed to the ABCD lower correlation on BHRCS (r = 0.56). In contrast, the influence of the age distributions was reflected more directly on MAE indicators, where the ABCD model presented the best performance on BHRCS (MAE = 1.47), followed by ADHD-200 (MAE = 2.17) and ABIDE-II (MAE = 2.26). Therefore, MAE indicators seem to be more influenced by the tendency of models to predict values nearby the center of its training distribution. In this way, the MAE is better (lower values) on datasets whose center of the distribution is closer to the center of the distribution of the training set. Conversely, the correlation (in cross-dataset evaluations) appears to be more influenced by the sample size and confounders (of the training set), and by the similarities between the images’ input features of the training and test data.

There were other interesting findings from the analyses of the most representative ROIs from models evaluated in distinct datasets (see Fig. 4). The top ROIs’ list from a model trained in one dataset was distinct from the ROIs’ lists of models trained in other datasets. This could be due to the optimization process (i.e., the training phase), where the model is pushed to learn features that best explain the target given the training data. As the training data distribution is distinct from one dataset to another (see Fig. 1), the most representative learned features would be the ones that best describe the data variability (i.e., age, sex, and mental health conditions). The more distinct the datasets are, the more different the features learned by each model will be, producing different lists of the most representative ROIs for each dataset. In contrast, the representative ROIs had little to no variability when a given model was evaluated against out-of-sample datasets. The list of the top 10 ROIs from a trained model was almost invariable when evaluated on out-of-sample distinct datasets (see Fig. 4). This is because trained models employ the same fixed parameters to assess any dataset. Therefore, the few differences in the lists of ROIs were due to the variability of the evaluated data.

The models trained from different datasets have the most distinct representative ROIs. When we evaluated these models against out-of-sample datasets, their representative ROIs remained nearly the same. Nevertheless, these distinct models can predict age from out-of-sample datasets with statistically significant performance. Moreover, these structural changes are enough to estimate aging by different models whose learned features are based on different sets of representative ROIs (see Figs. 2, 3, and 4).

The capability to estimate age (within- and inter-datasets) from models with distinct representative ROIs (see Fig. 2, 3, and 4) suggests that structural changes are distributed throughout the brain during neurodevelopment. This finding supports previous longitudinal studies, which found that GM and WM volumes change from childhood to adulthood^44,45^. Neural development involves highly coordinated and sequenced events characterized by both progressive (myelination) and regressive (synaptic pruning) processes^46^. A two-year-old child can have 50% more synapses than an adult^47^. The synaptic pruning process reduces the number of synapses in a regionally and temporarily specific manner, resulting in more efficient connections^46^. Simultaneously, myelination generates a protective sheath around nerve axons, facilitating the speed and efficacy of neural communication^48^. In other words, synaptic pruning and myelination processes affect the GM and WM densities of distinct ROIs at different rates during neurodevelopment^45,46^. Therefore, the divergences in ROIs’ representativeness for models trained from different datasets agree with previous neuroscience knowledge.

All except the ABCD models presented representative ROIs on the cortical surface. In contrast, ABCD models focused mostly on subcortical regions, specifically in the midbrain and pons (see Fig. 2 and 3). Even the BHRCS models, whose datasets have demographics similar to ABCD; focused on completely different ROIs than the ABCD models. Again, a possible explanation for these differences could be the distinct and nonlinear rates of neurodevelopment in each brain region^45,46^. The midbrain and pons (focused by ABCD) embody a primitive role, controlling sensory and motor functions, including elements of the visual and auditory system^49^. Furthermore, three of the four major dopaminergic tracts originate in the substantia nigra of the midbrain^49^. Whereas BHRCS models focus on the sparse cortical regions, more specifically, on the right lobe, and the temporal, orbital, parietal, angular, and inferior frontal gyri. According to Gogtay et al.^45^, who analyzed brain maturation from childhood to adulthood, phylogenetically older brain areas mature earlier than that newer ones. More complex brain regions tend to mature after the more primitive ones^45^. This could also have influenced our results, as the datasets had different distributions of subjects in distinct stages of brain maturation.

The models’ failure to detect ASD, ADHD, and dimensional psychopathologies assessed by CBCL indicate that the structural alterations from these conditions are subtle and heterogeneous^8,50^ enough to not be captured by CNNs trained with sMRI from large datasets. In psychiatric disorders, large and heterogeneous data samples tend to deliver high confidence and generalization power; however, they also lead to low accuracy^43^, possibly affecting our results. Another potential constraint is related to the capacity of the CNN to internalize complex long-range relationships of input features. In this case, a possible approach could be the use of transformer-based normative models^50,51^. Transformers’ attention mechanisms model the dependency of input features without regard to their distance, enabling the acquisition of complex long-range relationships^50^. Moreover, modeling TD subjects to detect psychiatric conditions based on deviations from normality appears to be a good strategy to circumvent the issue of structural heterogeneities in psychopathology.

Despite the surprising generalization capacity of the age models to estimate out-of-sample datasets, given they were trained from datasets with diverse demographic variations (especially for the ABCD and BHRCS age ranges), the results should be interpreted cautiously. First, a significant performance loss can occur when estimating subjects with distinct demographics from the ones used for training. The more different the subjects are from the training demographics, the greater the performance loss. Second, the representative ROIs for the models’ decision-making were strictly specific to the population used during training. Thus, small demographic differences in the training sample can lead trained models to focus on completely different brain regions. Therefore, it is risky to make assumptions beyond the characteristics of the population used for training the model. Fourth, the confounders present in training data can bias the model during the learning process. Therefore, instead of learning generalizable features, the model can learn by the cofounders. This scenario causes the model to lose generalization power when it is exposed to non-confounded examples.

Keeping these limitations in mind, the models trained to estimate age had a satisfactory performance, presenting almost identical brain ROIs in out-of-sample dataset evaluation. However, the models could not adequately learn to estimate the brain structural differences produced by ADHD, ASD, and dimensional psychopathologies. Larger longitudinal samples are expected to provide better estimates. However, the complexity of psychiatric symptoms and syndromes may not be achievable through structural imaging via supervised CNN, during adolescence. In adolescence, many psychiatric symptoms are starting to emerge or are in the early stages, making their detection even more challenging.

## Data Availability

The datasets used in this study were obtained from two public datasets: the Autism Brain Imaging Data Exchange II (ABIDE-II) and Attention Deficit Hyperactivity Disorder (ADHD-200); and from two datasets that required authorization: Adolescent Brain Cognitive Development (ABCD) and Brazilian High-Risk Cohort Study (BHRCS). ADHD-200 and ABIDE-II can be downloaded from the NeuroImaging Tools & Resource Collaboratory Image Repository, after free registering and login, from the following download links, respectively: https://www.nitrc.org/ir/app/template/XDATScreen_report_xnat_projectData.vm/search_element/xnat:projectData/search_field/xnat:projectData.ID/search_value/adhd_200, and https://www.nitrc.org/ir/app/template/XDATScreen_report_xnat_projectData.vm/search_element/xnat:projectData/search_field/xnat:projectData.ID/search_value/ABIDE_II. For ABCD and BHRCS datasets, application and consortium approval of an NDA form are required. The data were collected and made publicly available according to the guidelines, and approval was provided by the local ethics committee for each project. Detailed information on these datasets and their acquisition parameters can be retrieved from ABIDE-II (http://fcon_1000.projects.nitrc.org/indi/abide/abide_II.html), ADHD-200 (http://fcon_1000.projects.nitrc.org/indi/adhd200/), ABCD (https://nda.nih.gov/abcd), and BHRCS (https://osf.io/ktz5h/wiki/home/).
