# Publisher Correction: Generalizability of 3D CNN models for age estimation in diverse youth populations using structural MRI

**DOI:** 10.1038/s41598-023-35101-y

**Published:** 2023-05-17

**Authors:** Sergio Leonardo Mendes, Walter Hugo Lopez Pinaya, Pedro Mario Pan, Andrea Parolin Jackowski, Rodrigo Affonseca Bressan, João Ricardo Sato

**Affiliations:** 1 Center of Mathematics, Computing, and Cognition, Universidade Federal Do ABC, Rua Arcturus N. 03, São Bernardo Do Campo, SP 09606-070 Brazil; 2 Department of Biomedical Engineering, King’s College London, London, SE1 7EH UK; 3 Escola Paulista de Medicina, Universidade Federal de São Paulo, R. Maj. Maragliano (UNIFESP), 241—Vila Mariana, São Paulo, SP 04017-030 Brazil; 4 Department of Education, ICT and Learning, Østfold University College, Halden, Norway

Correction to: *Scientific Reports* 10.1038/s41598-023-33920-7, published online 27 April 2023

The original version of this Article contained errors in the Materials and methods section, under the subheading ‘Software and hardware specification’, where links to used resources were omitted and the present link was incorrect.

“The sMRI preprocessing was done through the SPM12 v7771 software. All further steps used Python 3.8.5 and Tensorflow 2.4.0. The machine learning experiments were performed on an NVIDIA DGX-2 server, within a Docker virtual machine containing 4 CPUs @2.7Ghz and 1 GPU TESLA V100-SXM3-32 GB. All source codes are available at Github (https://docs.nvidia.com/deeplearning/frameworks/tensorflow-release-notes/rel_21-03.html).”

now reads:

“The sMRI preprocessing was done through the SPM12 v7771 software (https://www.fil.ion.ucl.ac.uk/spm/software/spm12/). All further steps used Python 3.8.5 and Tensorflow 2.4.0 (https://docs.nvidia.com/deeplearning/frameworks/tensorflow-release-notes/rel_21-03.html). The machine learning experiments were performed on an NVIDIA DGX-2 server, within a Docker virtual machine containing 4 CPUs @2.7Ghz and 1 GPU TESLA V100-SXM3-32 GB. All source codes are available at Github (https://github.com/SergioLeonardoMendes/3dcnn_smri_generalization).”

The original Article has been corrected.

